# Comparison of the shear bond strength of RMGIC to a resin composite using different adhesive systems: An *in vitro* study

**DOI:** 10.4103/0972-0707.66716

**Published:** 2010

**Authors:** Varun Arora, M Kundabala, Abhishek Parolia, Manuel S Thomas, Viveknanda Pai

**Affiliations:** Department of Conservative Dentistry and Endodontics, Manipal College of Dental Sciences, Mangalore, Karnataka, India

**Keywords:** Sandwich technique, Self-Etch adhesive, Total-etch adhesive

## Abstract

**Aim:**

This study evaluated and compared the role of newer dental adhesives to bond composite resin to the resin modified glass ionomer (RMGIC) liner.

**Materials and Methods:**

Thirty-six specimens were prepared on acrylic blocks, with wells prepared in it by drilling holes, to retain the RMGIC. The specimens were randomly divided into three groups of 12 specimens each. In group I, a thin layer of an adhesive, which was a Total-etch type (Adper Single bond-2), was applied between RMGIC and the composite resin. Ingroup II, a Self-Etch adhesive (Adper prompt-L pop) was applied, and in group III there was no application of any adhesive between RMGIC and the composite resin. After curing all the specimens, the shear bond strength was measured using an Instron universal testing machine.

**Results:**

The results were drawn and tabulated using ANOVA-fishers and Tukey’s statistical tests. The maximum shear bond strength values were recorded in group II specimens with the self-etch adhesive (Adper prompt-L pop), showing a mean value of 5.826 when compared to the group I adhesive-Total-etch type with a mean shear bond strength of 4.6380, while group III specimens, where no adhesive was used, showed a minimum mean shear bond strength of 2.8385. There was a great and significant difference between group I and group II (*P* value 0.003), whereas, both group I and group II showed a vast and significant difference from group III (*P* value 0 – 001).

**Conclusion:**

Hence, this present study concludes that application of Self-Etch adhesive (Adper prompt-L pop) in between RMGIC and composite resin increases the shear bond strength between RMGIC and the resin composites, as compared to the Total-etch type adhesives (Adper Single Bond 2), as well as, without application of the adhesive agent.

## INTRODUCTION

Glass ionomer cements (GICs)developed by Wilson and Kent in 1971, have certain properties that make them useful as a restorative material of choice.[1] These properties include physicochemical bonding to both enamel and dentin, release of fluoride over a prolonged period of time, and the same coefficient of thermal expansion as dentin.[2–5] However, these cements usually have somewhat inferior esthetic and abrasion resistance when compared to composite resin, thus limiting their use in high stress-bearing areas. This leads to the development of the so called ‘sandwich technique’ or ‘bilayered technique’.[6] In this technique, a glass ionomer in conjunction with a superficial layer of composite resin has been established as an effective means of combining the favorable properties of both the materials in a single restoration. By decreasing the bulk amount of resin used, this technique can also reduce the detrimental effect of polymerization shrinkage, which may result in microleakage and marginal gap.[7] In addition, it acts as a stress absorbing layer between the shrinking composite and the dentin.[8]

Glass ionomer cement, when used as a base or liner, not only reduces the volume of the composite, but also prevents the composite resin from bonding with the dentin, thereby restricting the adhesion of the composite resin to the enamel. The reduction in bonded composite surfaces decreases the configuration factor (C - factor) of the cavity.[9] As a result, the tensile stress generated by the polymerization contraction of the composite is also reduced.

The first laminated restoration used conventional autocure GIC, which developed a mechanical interlock between it and the composite resin. Previous studies have shown that an etching of the GIC was recommended to improve its bonding to the composite resin.[10–11] The matrix of the hardened GIC dissolved in the acid, resulting in a rough and porous surface. The bonding agent could penetrate into the surface irregularities and harden them to provide resin tags for bonding.

However, of late, the etching procedure has been rejected, because it leads to a decrease in the strength of the GIC.[12] Besides, dissolution acid etching may also introduce cracks in the GIC. Phillips R has stated that the set surface of the GIC may provide enough roughness to ensure a bond of the resin, and excessive acid etching may destroy the cement.[13] The bond strength between conventional GIC and the composite resin is reduced by the low cohesive strength of GIC and by minimal chemical bonding, due to the different chemical reactions of these materials.[14] The initial, slow, acid–base setting reaction leads to high moisture sensitivity, low early strength, and progressive loss of GIC, thus ending in failure of restoration.[15] Hence, a resin-modified GIC (RMGIC) has been introduced, which has been modified by the introduction of polymerizable functional groups in its composition. This imparts rapid curing and exhibits a command set when activated by light or chemicals via the methacrylate group, and protects the acid–base reaction from immediate water uptake. It allows the acid–base reaction to take its course even after light polymerization and overcomes the inherent drawback of the conventional GIC by showing higher early strength, less moisture sensitivity, and more resistance to solubility and disintegration. RMGIC also demonstrates improved mechanical and physical properties when compared with the conventional GIC.[16] It shows better cohesive strength and lower modulus of elasticity than the conventional GIC.[17] The bond strength of RMGIC to the tooth is better than that of the conventional GIC. It also exhibits a higher bond strength to the resin composite.[14,18]

A strong bond between RMGIC and the composite resin is an important factor for the quality of bilayered restoration. Etching of RMGIC is not required prior to the bonding of the composite resin.[19] However, application of resin bonding agents promotes the adhesion of the resin composite to both conventional GIC and RMGIC.[20] Moreover, hydroxyethylmethacrylate (HEMA) incorporated into the glass ionomer cement, forms a chemical bond with the resin of the composite.

There is limited literature on the bond strength of RMGIC to composite resin with adhesive agents in between. However, bonding agents have been seen to improve the wettabilty of GIC to help it adhere to the composite resin.[21] Studies have shown that bonding agents which demonstrate a high degree of wettability, low viscosity,[21] and low contact angle[22] achieve a better union between GIC and the resin composite. Newer adhesive agents have undergone various modifications, such as, changes in viscosity, modification of primers, addition of nanofillers, and so on, to improve the bond strength between the tooth and composite resin. However, there are very few studies conducted to compare the bond strength between RMGIC and the composite, with different adhesive agents applied on the RMGIC.

Hence, the present study was conducted to evaluate and compare the shear bond strength of RMGIC to composite resin, using different generations of bonding systems applied on RMGIC.

## MATERIALS AND METHODS

Resin modified GIC (Vitrebond 3M ESPE, St. Paul USA) was bonded to a resin composite (Filtek^TM^ Z-350 3M ESPE, St.Paul USA) by using two different bonding agents, a Total-etch adhesive (Adper^TM^ Single Bond 2-3M ESPE, St.Paul USA) and a Self-Etch adhesive (Adper^TM^ Prompt^TM^ L Pop^TM^ -3M ESPE, St.Paul USA).

### Preparation of the specimens

The thirty-six specimens used in this investigation were prepared by using acrylic blocks. A total number of nine acrylic blocks was prepared using a cuboidal aluminum mould, 50 mm / 60 mm in dimension, which was polished with 220, 320, and 400 grit carbide polishing paper. In each block, four wells of 8 mm diameter and 2.5 mm depth were prepared by drilling holes in it, to retain the RMGIC. Grooves were placed on the walls for increasing the retention. The wells were then filled with light cure GIC by mixing it according to the manufacturer’s instructions and covering them with glass plates to produce a smooth surface and to permit light for curing the material. It was then cured with a blue *light-emitting diode* (LED) curing light (Monitex, Taiwan) for 40 seconds, according to the manufacturer’s recommendation, to produce a final set. The glass plate was carefully removed to ensure that the glass ionomer surface was smooth and not pitted.

Specimens were randomly divided in to three groups of 12 specimens each, the groups were:

Group I: To the light cure GIC (Vitrebond^TM^) a thin layer of Total-etch adhesive (Adper^TM^ Single Bond 2) was applied according to the manufacturer’s instructions and cured, and then a cylinder of composite resin (Filtek^TM^ Z 350, 3M ESPE, St. Paul USA) was added and cured over the specimen.

Group II: Same as group I, but a thin layer of Self-Etch adhesive (Adper Prompt L Pop) was applied and cured over RMGIC.

Group III: Acts as a control group, no adhesive agent was applied between the light cure GIC and resin composite.

Immediately following this procedure a transparent plastic ring, 5 mm in height, with a 5.5 mm internal diameter, was centered over the resin-treated GIC, in the templates. The composite resin was condensed into a transparent plastic ring, using an incremental curing technique, above the RMGIC substrate, and all sides of the specimen were cured to ensure complete curing of the material. Following the curing the plastic ring was removed. All the procedures were conducted at room temperature. The bonded specimens were stored in distilled water at 37 ± 2°C for 24 hours, to stimulate the oral cavity, until the specimens underwent the shear bonding test. The shear bond strength was measured by shearing of the bonded specimens on an Instron universal testing machine (model H 206), using a cross head speed of 0.05 mm / minute. The shearing apparatus was constructed to grip the acrylic block, and a wedge blade system was designed to apply a shear force of approximately 0.1 mm on the adhesive interface. The readings were tabulated and subjected to statistical analysis using ANOVA, Fisher‘s test, and Tukey’s test. Mean and standard deviation were calculated for each group by using the ANOVA test and intergroup comparison was done by the multiple comparison test — Tukey’s test, which revealed a statistical significance among the groups.

### RESULTS

**Figure 1:**
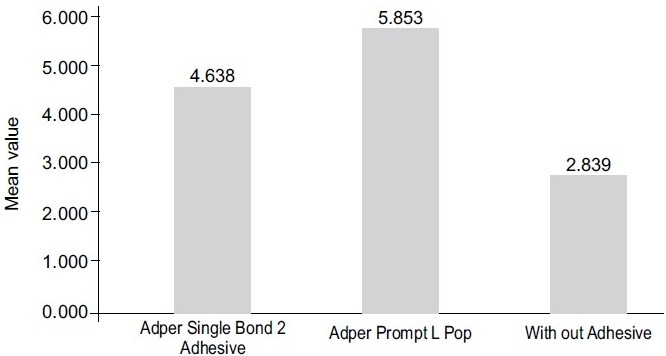
Mean value of shear bond strengths of experimental materials

The mean shear bond strength and standard deviations were calculated for each group [Table 1, Figure 1]; and analyzed using the ANOVA test. The maximum shear bond strength values were recorded for Group II, where the Self-Etch adhesive (AdperTM PromptTM L PopTM) was used, with the mean value of 5.85 MPa and a standard deviation of 0.962. On the other hand, Group III (without any bonding agent) displayed minimum shear bond strength, with a mean of 2.83 MPa and standard deviation of 0.6409, and Group I, with the Total-etch adhesive (AdperTM Single Bond 2 Adhesive) showed a mean shear bond strength of 4.63 MPa with a standard deviation of 0.826.

**Table 1:** Mean values (MPa) and standard deviation (SD) of the shear bond strength of the RMGIC bond to the composite by using different dentin adhesives

Groups	No.	Mean	SD	Minimum	Maximum
AdperTM Single Bond 2	12	4.6380	0.8369	3.5095	6.5235
Adhesive					
AdperTM PromptTM L	12	5.8526	0.9624	4.5004	7.4731
PopTM					
Without Bonding Agent	12	2.8385	0.6409	1.8580	3.6746

Intergroup comparison was conducted by using the multiple comparison test (Tukey’s test), which revealed a statistical significant difference among the groups [Table 2].

**Table 2:** Intergroup comparison and its statistical significance

Group	Group	Mean difference	*P* value
Group I-Total-etch adhesive (AdperTM Single Bond 2)	Self-Etch adhesive (AdperTM PromptTM L PopTM)	-1.214550	0.003hs
Group-II-Total-etch adhesive (AdperTM Single Bond 2)	Without bonding agent (Group-III)	1.799475	0.001 vhs
Group-I(AdperTM PromptTM L PopTM)	Without bonding agent(group-III)	3.014025	0.001 vhs

hs- highly significant, vhs-very highly significant

The Tukey’s test showed statistically high significant differences between Group I and Group II (*P* value 0.003), whereas, Group I and Group II in comparison to Group III showed a very high significant difference (*P* value .001).

## DISCUSSION

The primary objective of restoring any vital tooth is the prevention of pulpal insults.[23] The possible sources for adverse pulpal inflammation have been identified as thermal stimuli, chemical stimuli, and bacterial endotoxins.

The result of the present study has concluded that a Self-Etch adhesive agent, Adper^TM^ Prompt^TM^ L Pop^TM^, produces better shear bond strength to Vitrebond, a RMGIC, which is highly significant as compared to a Total-etch adhesive Adper^TM^, Single Bond 2, and to the group without any bonding agent. This may be due to the acidic pH of the Self-Etch adhesive. The acidic nature of adhesive agents makes the superficial surface of the GIC dissolve, thereby improving the bonding of GIC to the composite resin.[24] In addition to a low pH, the Self-Etch adhesive used in the present study has less viscosity compared to the Total-etch adhesive. A study by GJ Mount, 1989, has shown that a bonding agent having less viscosity shows a lesser contact angle to the surface, and results in better wettability, which helps in promoting a better bond between RMGIC and the resin composite.[22]

The present study is in agreement with other studies where the effect of surface treatments and storage methods on composite / GIC were evaluated,[25,26] where it was established that an application of a silane coupling agent over a non-etched GIC surface, followed by application of a bonding agent, demonstrated maximum shear bond strength.

In the present study RMGIC was used over the conventional GIC under composite resin restoration because RMGIC sets by an acid–base reaction and exhibits a command set when activated by light or chemical agents via the methacrylate group. RMGIC has also demonstrated a better bonding to composite resin than the conventional GIC.[17] This is due to a similar chemistry between RMGIC and the composite resin, which allows the strong bonding of RMGIC to composite resin. Both RMGIC and the resin composite are cured by a free radical initiator system, which provides a potential for the chemical bonding between these two materials.

## CONCLUSIONS

From the results of the present study it can be concluded that

- Application of bonding agents improves the wettability of glass ionomer cement to adhere to composite resin, thus promoting a strong shear bond between RMGIC and the resin composite.
- Moreover, the application of the Self-Etch adhesive between RMGIC and the composite resin showed significantly higher shear bond strength compared to the Total-etch adhesive.
